# Supplementary material for: The Impact of MOSE (Experimental Electromechanical Module) Flood Barriers on Microphytobenthic Community of the Venice Lagoon
Source: Microorganisms. 2023 Apr 3;11(4):936. doi: 10.3390/microorganisms11040936 (PMC10143115; doi:10.3390/microorganisms11040936)
Supplement: Supplementary file 1 [file microorganisms-11-00936-s001.zip › Figure S1.pdf]

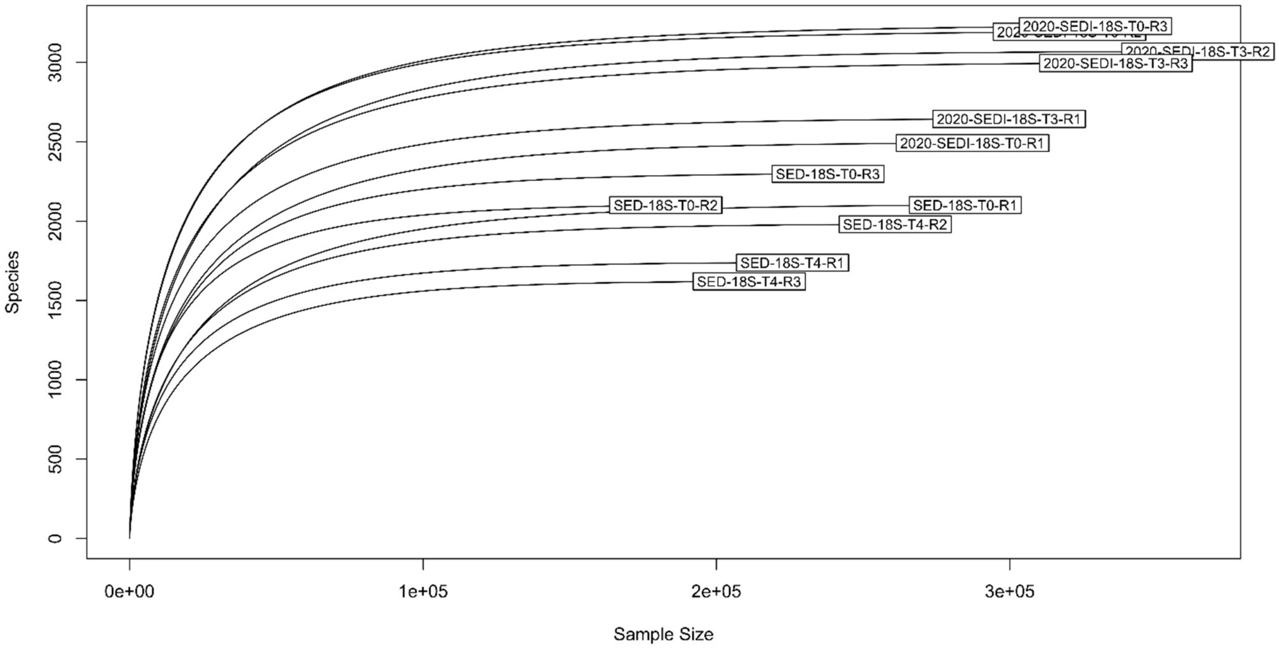


Figure S1. Rarefaction curves of the samples in terms of ASVs observed as the sample size increases up to the maximum number of available sequences.
